# Average findings of uric acid in blood in patients with gout with different categories of hyperglycemia

**DOI:** 10.1186/ar3633

**Published:** 2012-02-09

**Authors:** Ulugbek K Kayumov, Marif Sh Karimov, Nargiza A Abdukhakimova

**Affiliations:** 1 Tashkent Institute of Postgraduate Medical Education, Tashkent, Uzbekistan; 2 Tashkent Medical Academy, Tashkent, Uzbekistan

## 

Along with a huge amount of works about the importance of a metabolic syndrome in development of cardiovascular diseases, within last decade in the literature there was a series of reports on a pathogenetic role of this syndrome in formation and more serious current of some other diseases of an internal. In process of doctrine development about a metabolic syndrome [1], there was new data about existence at gout of various signs insulin resistance [2]. At the same time, there are insufficiently studied questions on a role of various categories of a hyperglycemia (a hyperglycemia on an empty stomach and a postloading hyperglycemia) in a pathogenesis and gout and hyperuricemia clinic.

## Method of the inquiry

120 males with gout at age 30-69 were examined to investigate the connection between different categories of hyperglycemia and level of uric acid in patients with gout. Gout was revealed on the basis of criteria of American Rheumatic Association. Glucose tolerance condition was revealed by carrying out standard test of glucose tolerance (TGT) with revealing of glycemia on an empty stomach, and also in one and two hours after taking 75 gr glucose by the examined patients.

## The results

According to the revealed findings average levels of uric acid in patients with gout with normal glucose tolerance had 531,56 ± 0,38 mcmol/l. With damaged glucose tolerance on an empty stomach and in two hours after glucose loading, levels of uric acid were more higher(658,18 ± 0,27 mcmol/l and 656,22 ± 0,34 mcmol/l correspondingly). At the same time on damaged glucose tolerance in an hour after glucose loading average level of uric acid was 501,16 ± 0,33 mcmol/l. We should draw attention that the difference of average levels of uric acid among people with disorders glucose tolerance on an empty stomach and in two hours after glucose loading was more differ from level of uric acid among people with glucose tolerance disorder in an hour after glucose loading (p*<0,05).

## Conclusion

According to these results we can come to the conclusion that the level of hyperglycemia has connection with existence in patients with hyperglycemia on an empty stomach and two hours after glucose loading. At the same time the problem about connection of uric acid level with hyperglycemia in an hour after glucose loading should be examined farther. Perhaps, that rising of glycemia level in an hour after glucose loading is a compensator mechanism in patients with gout.

**Table 1:** Average findings of uric acid in blood in patients with different categories of hyperglycemia

Categories of hyperglycemia	n	M	± m
Normal tolerance	13	531,56	0,38
Hyperglycemia on an empty stomach	11	658,18 *	0,27
Hyperglycemia after 1 hour	20	501,16	0,33
Hyperglycemia after 2 hours	76	656,22 *	0,34

* Note: in the table is shown the reliability of differences concerning an indicator in hyperglycemia group in 1 hour after loading a glucose.
